# Detecting the Activation of a Self-Healing Mechanism in Concrete by Acoustic Emission and Digital Image Correlation

**DOI:** 10.1155/2013/424560

**Published:** 2013-12-08

**Authors:** E. Tsangouri, D. G. Aggelis, K. Van Tittelboom, N. De Belie, D. Van Hemelrijck

**Affiliations:** ^1^Department of Mechanics of Materials and Constructions, Vrije Universiteit Brussel, Pleinlaan 2, 1050 Brussels, Belgium; ^2^SIM vzw—Program SHE, Technologiepark 935, 9052 Zwijnaarde, Belgium; ^3^Magnel Laboratory for Concrete Research, Department of Structural Engineering, Faculty of Engineering, Ghent University, Technologiepark Zwijnaarde 904, 9052 Ghent, Belgium

## Abstract

Autonomous crack healing in concrete is obtained when encapsulated healing agent is embedded into the material. Cracking damage in concrete elements ruptures the capsules and activates the healing process by healing agent release. Previously, the strength and stiffness recovery as well as the sealing efficiency after autonomous crack repair was well established. However, the mechanisms that trigger capsule breakage remain unknown. In parallel, the conditions under which the crack interacts with embedded capsules stay black-box. In this research, an experimental approach implementing an advanced optical and acoustic method sets up scopes to monitor and justify the crack formation and capsule breakage of concrete samples tested under three-point bending. Digital Image Correlation was used to visualize the crack opening. The optical information was the basis for an extensive and analytical study of the damage by Acoustic Emission analysis. The influence of embedding capsules on the concrete fracture process, the location of capsule damage, and the differentiation between emissions due to capsule rupture and crack formation are presented in this research. A profound observation of the capsules performance provides a clear view of the healing activation process.

## 1. Introduction

The use of expansive healing agents encapsulated into tubular capsules and actuated when damage occurs, appears to be one of the most promising autonomous healing systems in concrete research [1]. Van Tittelboom studied in detail different healing agents and encapsulation approaches and selected tubular glass capsules placed in couples filled with a two-component polyurethane-based healing agent and embedded into concrete elements. In more details, the healing mechanism is activated when crack formation ruptures the pairs of glass capsules and the two-component healing agent is released filling the crack volume. The polyurethane healing agent polymerizes when both agents come into contact. After a few hours of curing the intended repair is achieved [2].

Recently, the mechanical performance of the aforementioned smart material was investigated under different damage and loading conditions [2]. The recovery of cracked areas is confirmed under bending. In one of those studies, Acoustic Emission (AE) confirmed capsule breakage and was used to monitor crack formation and repair during two cycles of bending tests [3].

Preliminary results of our research on the Self-healing (SH) performance under bending load seemed promising [4], although obtaining accurate analyses on such a complicated material remains challenging. A clear indication of cracking damage and distinction from capsule fracture are required. Furthermore, profound analysis of the conditions under which SH activation occurs will improve the material manufacturing process. In this study, an attempt was made to monitor by optical and acoustic techniques the capsules performance during crack formation. The obtained results focus on the trigger mechanism of healing while the mechanical performance of the smart material system is also important.

## 2. Three-Point Bending Damage

**Figure 1:**
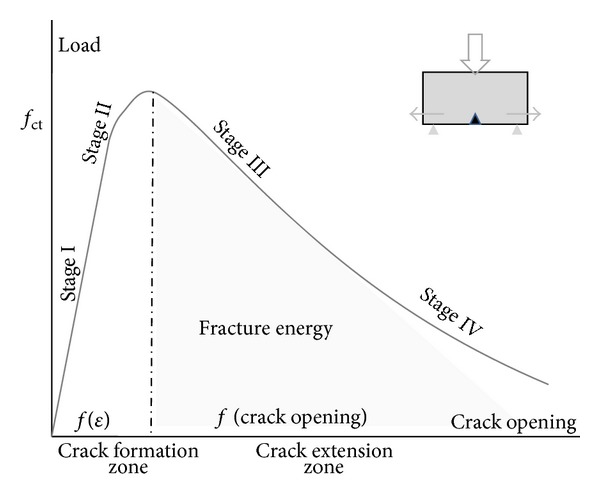
Fracture model of three-point bending crack opening test.

The study of materials damage, namely, fracture mechanics, provides analytical and experimental approaches in order to understand and prevent failure. Complicated multiple crack formation is initially simplified to a single notched crack analysis that provides the fundamental knowledge and crucial understanding of the damage process. Several methods approach the quasibrittle nature of plain concrete under mode-I bending fracture [5]. Common point between all is the five stages of crack formation under opening mode testing presented in the load versus crack opening curve shown in Figure 1. Initially, a linear elastic response of concrete (stage I) is obtained. Stage I ends when the stress applied reaches the concrete tensile strength and a crack forms. At that moment, the crack initiation point, namely, the crack tip, concentrates the critical stresses that govern the crack evolution (Stage II). Crack propagation occurs as the loading capacity of the material decreases and crack deformation evolves (Stage III). The plastic fracture process zone around the crack tip propagates following the crack path and enclosing crucial microcracks. The final bending fracture stage (Stage IV) states local failure when the limit crack opening width is reached [6].

The energy balance approach is a well-established fracture model that predicts the crack formation mechanism [7]. In more detail, during mode-I cracking part of the elastic stored energy is absorbed to create free damaged surfaces and another part is released due to fracture [8]. The key of the crack propagation mechanism is the unstable energy equilibrium that leads to energy release during fracture [9].

The material response will potentially differentiate due to the presence of embedded capsule. It is well known in the literature that irregularities in material composition can induce stress concentrations. In that direction, research has been done on the influence of aggregates size and nature on cracking response of concrete samples. In this respect, tubular capsules filled by healing agent acting as distortions may affect the cracking process. An analytical study of capsule breakage phenomena during concrete crack opening is required in order to clarify their impact on material fracture. The crucial stage of cracking at which capsule rupture occurs should be well defined since at that time healing repair is activated. More specifically, the healing triggering event should be characterized in time and accurately located within the material.

In this study, crack formation under three-point bending is investigated in the case of concrete samples with and without encapsulated healing material. Advanced optical and acoustic methods are used to monitor the performance of capsules and locate the healing activation. An analytical overview of acoustic signals captured by AE may provide experimental indications of capsules performance.

## 3. Materials

Plain concrete specimens were casted. The absence of reinforcement guarantees pure crack opening phenomena under bending. A water/cement/sand/coarse aggregate ratio of 1/2.07/4.28/4.52 was chosen. The maximum aggregate size was 13 mm.

**Figure 2:**
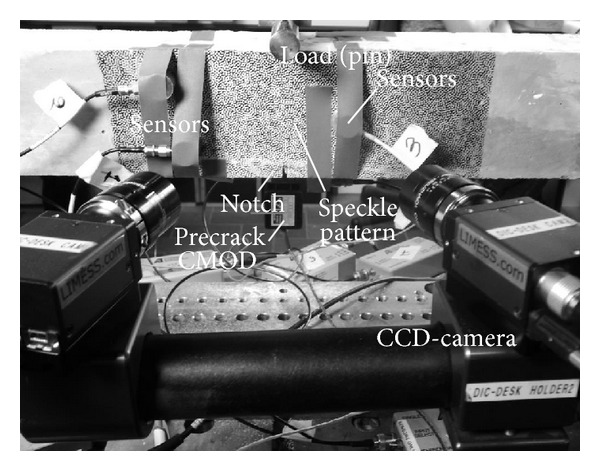
Experimental set-up.

After mixing the concrete was casted into wooden molds 840 mm long, 100 mm wide, and 100 mm high. At the bottom of the wooden mold a Teflon slice was positioned in order to create a notch in the concrete beams. The notch, shown in Figure 2, covers the width of the beam and its cross section is 3 mm wide and 10 mm high. The notch was created at the middle section of the beams and its presence was compulsory in order to induce crack formation at the middle of the beam. The sample dimensions were chosen according to the Rilem Technical Committee TC 50-FMC recommendation report on concrete fracture mechanics computations [10].

The beams were casted and consequently vibrated. Twenty-four hours after casting, the samples were demolded and stored under water for 14 days until testing time.

Reference samples did not contain any capsules in contrast to samples with self-healing properties within which couples of capsules were placed above the notch up to the neutral axis height.

**Figure 3:**
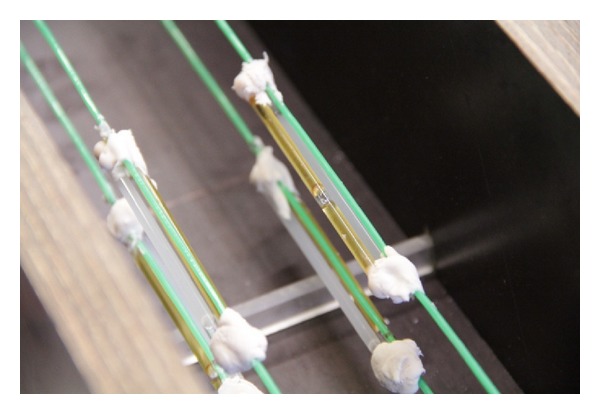
Series of capsules placed into the mold during casting.

Two-component polyurethane-based healing agent was embedded into borosilicate glass capillaries [1, 2]. The tubes, shown in Figure 3, are 50 mm long, the inner and outer diameters are 3.00 and 3.35 mm, respectively, and are sealed by glue at both sides. To prevent movement of the capsules during mixing, the capsules were attached to thin, poor in stiffness metallic wires crossing the length of the beam.

## 4. Methods

### 4.1. Three-Point Bending Configuration

The span between the test supports was fixed at 800.00 mm. An Instron load cell with a 20 kN capacity was used and the load was applied with a speed of 0.04 mm/min at the midsection of the sample.

A Crack Mouth Opening Displacement (CMOD) device, guided by EN 14651, was positioned over the notch to provide a precise display of the crack opening. Prior to testing, the CMOD gage was calibrated on 10.00 mm amplitude.

During testing, the load, the displacement, and the CMOD crack opening values were monitored and stored on a raw data file. Bending was applied until a crack opening of 0.40 mm was reached. Samples were subsequently unloaded, stored at room temperature for 72 hours of curing, and then reloaded following the same procedure.

**Figure 4:**
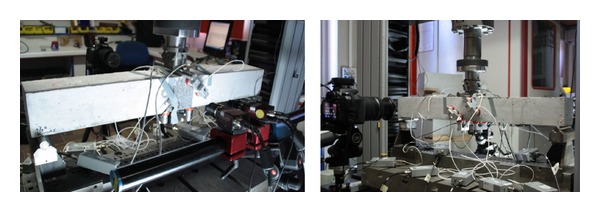
View of experimental setup configuration.

The present study focuses on the initial crack formation and capsule activity. Digital Image Correlation (DIC) and AE full-field monitoring of the testing process crucially contributes to this goal. Indicatively, the experimental setting up combining the different techniques is shown in Figure 4.

### 4.2. Digital Image Correlation Analysis

DIC is an optical measuring technique that determines displacement fields and strain distribution profiles of a sample when movements due to loading occur.

A 2-cameras system fixes an orthogonal coordinate system as shown in Figure 4—*X*, *Y*, *Z* (measured in mm or pixels)—and observes the random black-white speckle pattern painted at the surface of the sample. The stereoscopic cameras set-up captures periodically (every 3 seconds) high-resolution images and provides a series of virtual strain gauges fully covering the fractured area. Stereo-correlation between the images allows calculation of the sample position and deformation—*U*/*V*/*W* (measured in mm or pixels)—as crack opening occurs. Local derivative calculations provide Lagrangian strain tensor profiles—*e*_*xx*_/*e*_*yy*_/*e*_*zz*_ (measured in *μ*strain, mm/mm, or %)—across the entire surface under analysis. Also, the DIC software is able to calculate the maximum principal strains *e*_1_ and *e*_2_ obtained by the *e*_*xx*_ and *e*_*yy*_ combinations for *e*_*xy*_ = 0.00 [11].

The cameras were positioned next to the INSTRON device and aligned on a metallic cylinder attached to a tripod. The DIC set-up was balanced and both cameras were oriented perpendicular to the concrete sample. The set-up parameters are shown at Table 1 and Figure 4.

Settling the focus of the cameras, a calibration table was positioned at the sample region, and a calibration analysis was finalized by the DIC software giving a projection error (standard deviation error) less than 0.035. Then, the sample was placed back to the testing position, the CMOD device was attached to the beam and balanced, the load cell was reset, and three to four pictures were captured to insure that correlation could be done by DIC software for this set-up of testing.

### 4.3. Acoustic Emission Analysis

Acoustic Emission analysis can be used to detect crack initiation and to monitor crack propagation. Apart from that, the AE technique can be applied to locate and outline capsule rupture and to notice the interaction between concrete and capsule material [12–15]. As innovation in this research, the analysis of crack opening by AE is combined with the AE activity of embedded encapsulated healing agent.

Eight AE resonant R-15 PZT transducers were placed on both sides and the bottom of the sample. Stripe elastic tape and holding clamps with a magnetic base are applied to attach the sensors. The sensors named Channel 1, 2, 3, 6, 7, 8 were positioned antisymmetrically with respect to the depth of the beam at the sides of the beam. Channels 4 and 5 were placed symmetrically at the lower surface with respect to the notch. The sensors were attached to the surface by use of Vaseline coupling sealant and captured elastic waves arriving at the surface of the beam with amplitudes higher than 45 dB (AE threshold). Thereafter, gain of 40 dB was provided before the sensors activity reached a 32-bit digital signal processor. The analog bandwidth, the waveform, and timing parameters are shown in Table 2.

Hit-driven analysis was applied and it provided several waveform AE features. Some of them were selected representing and illustrating the damage process (amplitude, energy, duration, rise time, average frequency, counts, and counts to peak).

**Figure 5:**
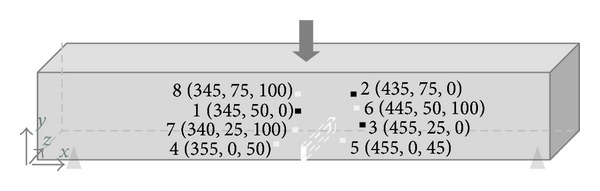
Coordinates system setup and sensors location.

Apart from the AE hits evaluation, the origin of the signal can be detected in 3D by the source location algorithm. Being aware of the wave velocity (as calculated by the pencil lead break test) and sensor position (as given in Figure 5), the source location can be determined by the time required for the wave to travel through the material and reach at least four sensors [16].

Taking into account the signal magnitude degradation due to attenuation effect, the localization analysis setup is given at Table 3.

## 5. Experimental Results

**Figure 6:**
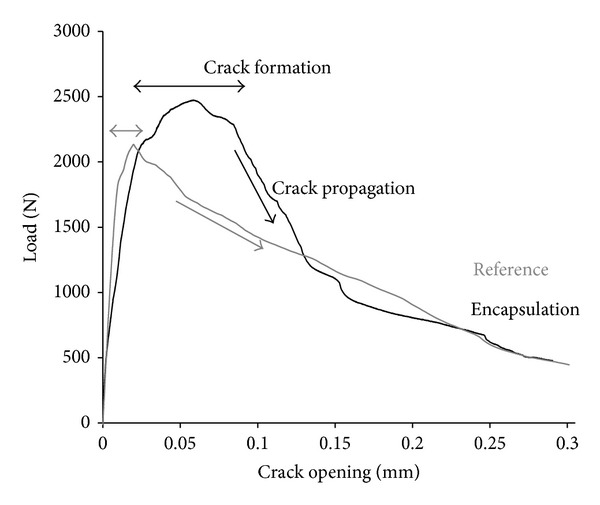
Load-crack opening graph for a representative sample of the reference and encapsulation series.

The evolution of the crack during performance of the three-point bending test provides primary information of the behaviour of samples with embedded capsules compared to reference samples. The load-crack opening displacement (measured by the CMOD device at the bottom of the beam) curves shown in Figure 6 are derived from a representative sample of the reference group of four beams (i.e., reference series) and another of the group of six beams into which healing system is embedded (i.e., encapsulation series).

**Figure 7:**
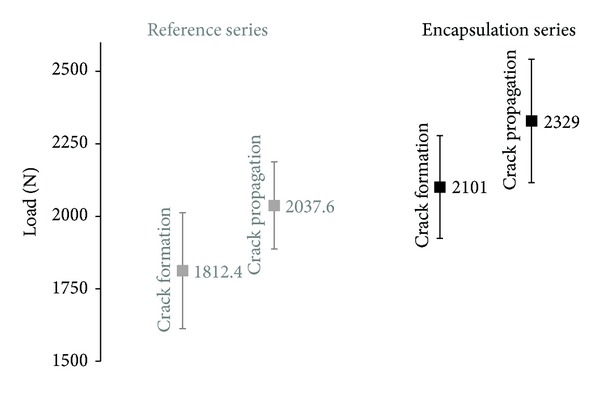
Material properties under bending: mean and standard deviation distribution for the sum of the beams.

Material properties of both test series as the initial stiffness, the load at crack formation, the maximum load, and the post peak load evolution can be derived from the graph in Figure 6. The values accounting for fracture response are quantitatively presented in Figure 7.

It is concluded that the initial stiffness (represented by the slope of the first part of the curves shown in Figure 7) is relatively similar for both series. As expected, the addition of capsules does not influence the linear elastic behaviour of the concrete matrix. On the other hand, the load at the moment of crack formation is slightly increased in the case of encapsulation test series indicating potential influence of capsules on the loading performance of the samples [1]. Further on, the encapsulation series maximum load obtained is significantly elevated compared to the reference one. The behaviour of the encapsulation series during crack propagation is greatly diversified and in combination with the extended fracture energy release offers a clear evidence of the effect of capsules on the loading response and resistance of the concrete samples.

In the literature, different fracture models analyze the crack path morphology and crack tip performance under bending. For instance, the fictitious crack model, generalizing several quasibrittle fracture models, defines the linear elastic zone of loading as the stage at which loading depends on the strain capacity of the material and defines the postpeak zone as the stage at which crack opening controls the decrease in load. Graphically, the fracture theory splits the loading graph into two zones as shown in Figure 1. The energy release due to crack extension is analogous to the area under the curve of Figure 1.

Microcrack formation around the crack surfaces, stress concentration in front of the crack tip, and local redistribution of strains due to crack propagation are the crucial factors of the evolution of crack opening.

Apart from the aforementioned damage principle, it is assumed that encapsulated material probably gives enhanced energy release due to the capsule breakage process. The fracture process at the concrete matrix-capsule interface and sliding of the capsule leads to microcrack formation or debonding effects that may justify the enhanced crack extension energy release. Determination of the location and time of capsule breakage during bending is required to obtain a definite explanation.

### 5.1. Crack Opening Activity Monitored by DIC: An Overview of Damage

**Figure 8:**
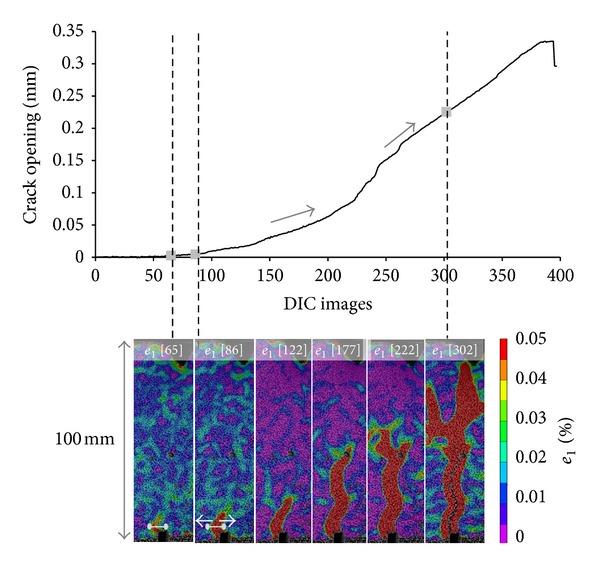
DIC monitoring strain and crack opening during bending.

DIC strain and deformation profiles provide an overview of the damage evolution as the crack forms, propagates, and opens till failure. In Figure 8 a progressive view of the crack morphology is presented and the continuously rising crack opening quantitatively measured at the bottom of the sample is shown. The position, at which the crack opening was calculated, is shown with a white mark in Figure 8.

In agreement with the fracture models analyzed above, at first, limited strain concentration appears at the edge of the notch. The principal—*e*_1_—strain profile of DIC image 65, corresponding to the end of Stage I of fracture indicates, the damage initiation before crack opening occurs. At the beginning of the experiment, the crack opening calculated at the concrete area above the notch remained negligible until that moment at which strain concentration locally appears, as shown at the strain profile *e*_1_  [65] graph of Figure 8.

The principal strain of DIC image 86 defines the end of crack formation and the beginning of crack extension. Crack initiation is confirmed by the limited crack opening shown at the crack displacement graph in Figure 8.

Subsequently, upon completion of stage II (visualized in images 122, 177, and 222), the crack propagates and gets wider in two different opening rates, indicated by arrows at the graph of Figure 8 up to the DIC strain profile of image 302 at which cracking propagates till the highest region across the height of the sample. That moment defines the end of fracture Stage III. Further on, mainly crack widening occurs leading to final failure (Stage IV of fracture).

It is shown on the strain profiles that the crack propagates above the precrack groove and reaches the top region of the beam. Capsules positioned orthogonal to the crack path are expected to break under bending. DIC optical observation of the cracked region leads to the next analysis focussing on the detection of tube breakage.

### 5.2. Acoustic Emission Energy Classification of Damage

**Figure 9:**
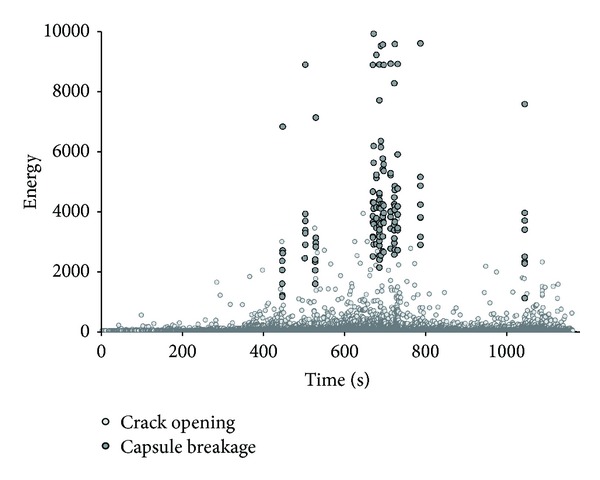
Hits energy activity during testing-detection of capsule breakage high energy hits.

An Acoustic Emission energy-based analysis was performed in order to distinguish capsule breakage from other damage events [17, 18]. Van Tittelboom et al. differentiate AE events due to capsule breakage, that provide high energy hits, from matrix cracking which releases lower energy-carrying hits [3]. Based on that ascertainment, in Figure 9 the energy of all AE hits captured during testing is shown. Analyzing the energy range, the presence of brittle instantaneous capsule breakage events is confirmed.

In more detail, every time a glass tube breaks, each of the AE sensors captures a waveform, represented by a set of descriptors analyzed in this research. Thereby, the AE hits energy derived from the area under the waveform shape is indicative to the released fracture energy and its magnitude and general characteristics are indicators of the damage mode. High energy hits correspond to discrete events of capsule breakage and are well differentiated from the rest of the AE events. Only in case of capsule breakage, the energy received by all the eight sensors placed at surface of the beam exhibits values ranging from 2000 to 12000 depending on the receiver's sensor distance from the event location (the energy of hits is relatively low in case of sensors placed far from capsules region and higher in case of sensors standing closer to the capsule fracture). Any other AE activity belongs to the second cluster as plotted in Figure 9 and its source is investigated below.

Regarding the bending mode crack formation, AE energy analysis may not differentiate the stages of crack evolution and the influence of capsules in the crack plane and the fracture process zone of the surroundings. Material damage optically based observation by DIC points out any diversity due to encapsulation.

### 5.3. Detecting the Conditions under Which Capsule Breakage Activates Healing

**Figure 10:**
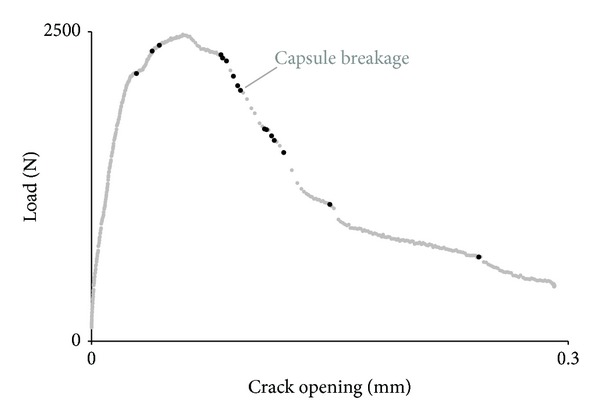
Capsules breakage events localization by AE at the load-crack opening graph.

Localization of capsule breakage was successfully done by synchronizing the AE activity with the Instron load data and DIC periodically captured images. Subsequently, the loading stage and the coordinates in three directions for each AE capsule breakage event were detected. Capsule damage events are plotted on the already discussed load-crack opening graph and are shown in Figure 10. Primary capsule rupture is coincided with crack initiation. Further, crack formation and propagation lead to a series of capsule breakages well-distributed in the postpeak region of the load graph.

Consistent with the graph in Figure 10, the fact that capsules break after crack initiation is verified. Stress concentration at the notch during Stage I is not sufficient to rupture the glass tubes. On the contrary, primary deformation, visualized at DIC image 86 strain profile of Figure 9, is the trigger mechanism of autonomous crack healing. Furthermore, it is concluded that the extended crack formation stage for the encapsulation series compared to the reference series appears due to breakage of the capsules, as shown in Figure 8. The release of stresses concentrated at the interface between the tubes and the concrete matrix and the undistributed crack propagation due to capsule irregularities leads to the steep decrease in load as capsule rupture occurs.

**Figure 11:**
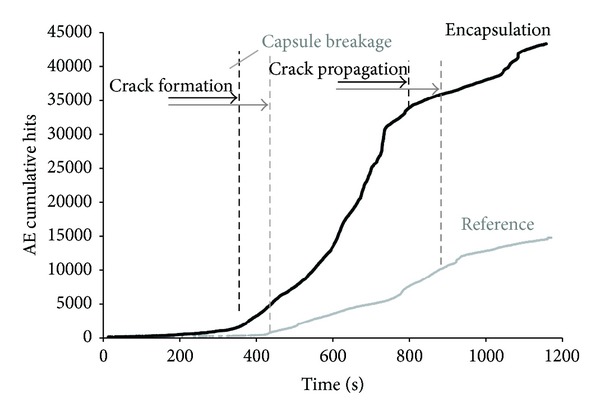
AE cumulative hits activity during testing for reference and encapsulation series.

For further information, the sum and the distribution of AE hits during testing for both series is included in this study. An augmentation of the number of hits due to capsule embedment is observed at the cumulative hits graph as presented in Figure 11. In more detail, linear loading response provides limited AE hits activity in both series. Crack formation leads to energy release due to crack deformations and gradually the number of AE hits increases. It is noticed that AE hits activity initiates earlier in the case of encapsulation beams. Local heterogeneity and stress concentration at the region of capsules placed above the notch can justify the prior AE activity. Building up of the fracture zone and crack expansion signifies the sharp rise of hits for both series of testing. In total, the AE activity of the encapsulation series at the end of the crack propagation stage is three times more extended than for the reference series. AE activity of the reference beam is related to crack opening and microcrack formation in the vicinity at both damaged sides of the beam. Well-distributed concrete material components may not arrest the crack propagation as it is shown already at the featureless postpeak region of the load-crack opening curve in Figure 6. On the other hand, crack opening of encapsulation series is a combination of complex fracture phenomena. Capsules placed above the notch create stress concentration and increase of the energy required to form cracks. Furthermore, capsule rupture involves the high energy release due to brittle fracture of the tube, potential debonding at different places across the capsule length, and friction or sliding forces between concrete and glass tubes that provide continuous AE activity and constant low energy release. The fracture activity is marked also at the energy-based analysis graph discussed already in Figure 9.

### 5.4. Further AE Postprocessing Analysis-Location of AE Capsule Events

**Figure 12:**
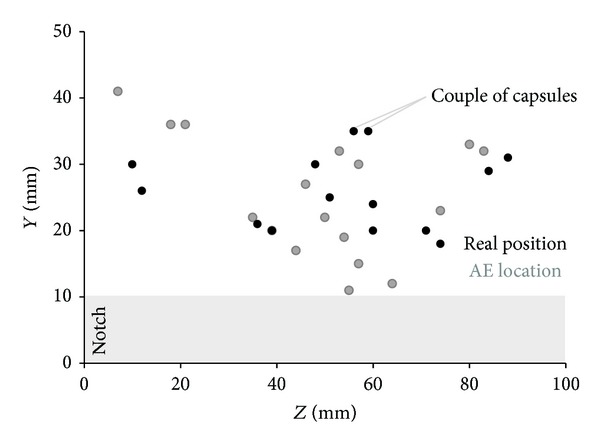
Real and AE located capsule breakage position at the crack plane.

After completion of the two-cycle bending test, the sample is broken in two pieces and the capsule coordinates were determined. Probable shortage of precise AE analysis can be assessed when the real location of capsules measured at the crack plane is compared to the coordinates given by the 3D localization algorithm. In Figure 12 the two-dimensional (*Y*-*Z* coordinates in mm) map of capsule breakage events at the crack plane is presented. In different gray-scales the actual and AE calculated location of the capsules is given. Slight variations—less than 8.00 mm—in the results observed fall within the accuracy limits of AE. In general, accurately located capsule rupture validates the localization approach and AE seems as a promising method to detect embedded healing systems in concrete.

Regarding the loading response of the sample, it is worth noticing that a big amount of capsule couples stand 20–30 mm above the notch. The heterogeneity of that zone justifies the accumulate stress applied at the postpeak stage of loading to break the concentrated glass tubes and let the crack propagate.

Careful observation of AE localization results points out a conflicting note. Inside the sample 7 couples of glass tubes are placed which entails that 14 capsule breakages are expected. Apparently, the AE algorithm locates 18 individual capsule breakages in the case of the beam analyzed. For all the other beams of the series 17–25 events of capsule rupture are captured. It is reasonable to conclude that some capsules break at multiple places as cracking propagates. Apparently, breakage of a tube on a specific point leads to local strength loss. Local discontinuity redistributes the loading across the capsule surface and rearranges microcrack effects on the concrete zone that covers the tube. In any case, the loading capacity under bending is maintained away from the damaged spot. Thereby, a capsule may break more than once in different sections as soon as the crack deformation applies unsustainable loading on them.

**Figure 13:**
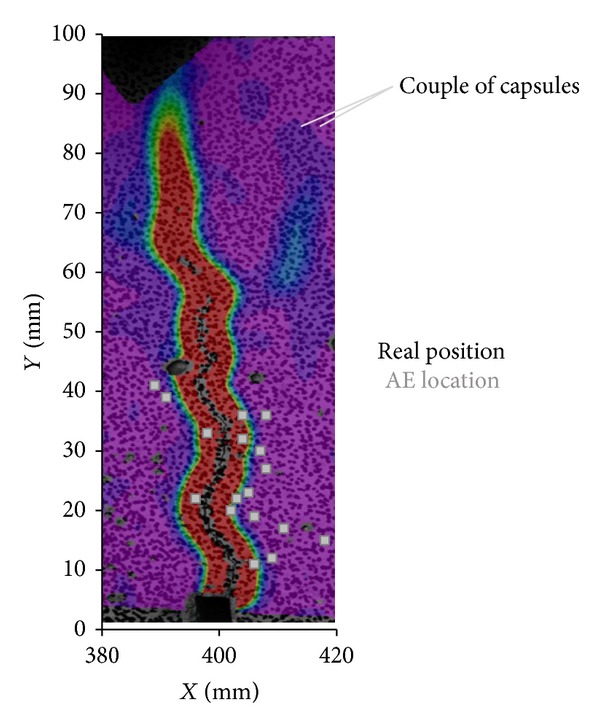
AE capsule location across the length of the beam and crack opening DIC fracture profile.

Apart from the crack plane observations, the capsule breakage phenomena are also located across the length of the sample. In Figure 13 the capsule rupture events are projected at the surface of one side of the beam. In parallel, a DIC strain profile is fixed to the same coordinate system and shown as background plot of the analysis. It is concluded that capsules do not always break at the crack plane. It should be kept in mind that the DIC monitors surface strain profiles and that crack propagates in different paths across the thickness of the sample avoiding aggregates and capsule constraints.

This observation confirms the presence of substantial fracture mechanisms acting at the limits of cracking damage and further away into the concrete matrix. As the crack forms, concrete microcracks in the vicinity of damage and the glass tube receive the fracture energy released and redistribute the strain formation.

### 5.5. Further AE Postprocessing Analysis: Measuring by DIC and AE the Crack Opening Whereby Capsules Break

**Figure 14:**
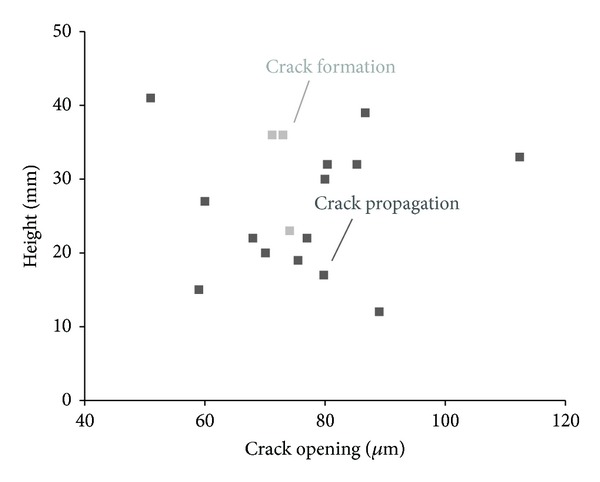
Local crack opening during capsules breakage.

Accurate AE events localization and DIC deformation profiles were combined once again to quantify the crack opening which was sufficient to fracture the glass capsules. In practice, knowing the coordinates of capsule failure events and the loading stage at that time, full-field DIC displacement recording provides the displacement at different heights along the crack improving the measured CMOD values of crack opening. The associated results are presented in Figure 14. According to the damage evolution, it was chosen to allocate a different color to the capsule breakages that appear at the stage of crack formation and those during crack propagation. For all cases, the crack opening ranges from 50 to 90 *μ*m proving that low crack opening may sufficiently trigger capsule breakage. The location of the tube at upper positions across the height of the crack plane appears not to delay the rupture nor to require higher values of crack opening. In parallel, it is observed that similar loading and stress conditions lead to capsule fracture throughout the entire bending test. Widening of the crack during loading does not further damage the tubes and after tube breakage the release of the agent should successively fill the crack plane.

### 5.6. Further AE Postprocessing Analysis: Clustering of AE Features

Clustering of cracking and capsule breakage AE hits according to their energy released, which was shown and discussed above, is the first approach in order to differentiate the global bending opening from local and instant capsule fraction. Retaining the energy clustering of hits, several other AE features are classified.

**Figure 15:**
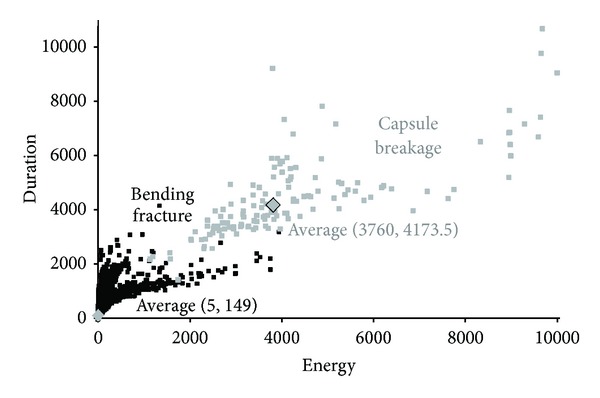
Clustering based on energy and duration features.

To start with, the signal duration was investigated since this is together with the energy values are the most representative waveform strength indicator. The plot of hits energy and duration captured during testing provides a clear evidence of clustering between material fracture and capsule breakage as shown in Figure 15.

**Figure 16:**
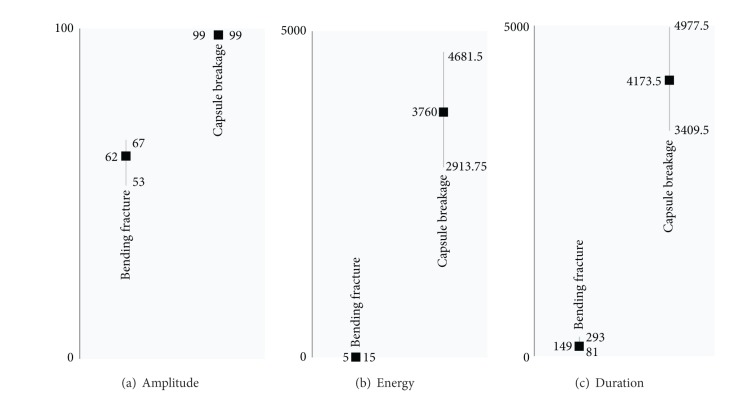
Clustering of AE features-part 1.

In parallel, a concise summary of other AE features is presented in Figure 16. Energy and duration classification is reviewed providing the mean, the first, and the third quartile of their values. Box plots of median and spread values are also prepared for the amplitude population of hits. Even in this case, the amplitude of capsule breakage significant differentiation is evident.

**Figure 17:**
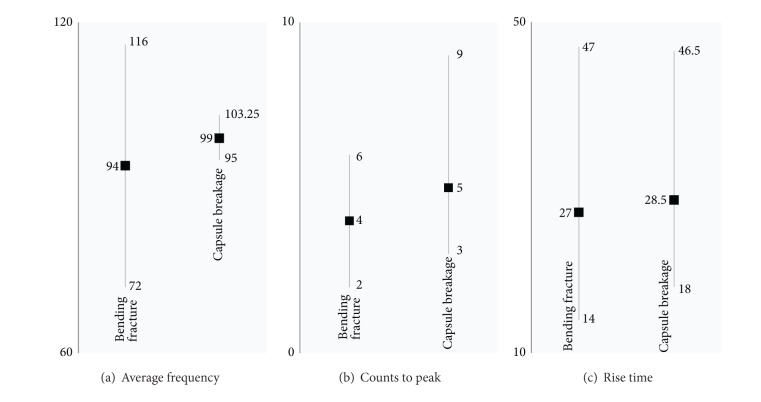
Clustering of AE features-part 2.

In contrast, the classification of hits based on average frequency, counts to peak, and rise time is not as straightforward as above. The hits of capsule breakage are masked by the opening mode of damage as shown in Figure 17. It is worth noticing that the average frequency values of the second cluster (capsule breakage) are well concentrated around the mean value. On the other hand, the different fracture phenomena falling in the first cluster show a wide spread frequency range.

## 6. Conclusions

The aim of this study, to detect the mechanism that activates autonomous crack healing of concrete beams under bending, is achieved. The challenging task is accomplished by optical and acoustic monitoring of the damage evolved. Acoustic Emission provided an integrated overview of capsule brittle fracture. The location of failure inside the material and identification of the conditions (loading, time, crack width, and fracture evolution) under which capsule breakage occurred were done by analyzing the hits activity captured by eight sensors attached to the concrete surface.

Classification of matrix damage in combination with interplay to the glass tubes interface and the capsule breakage was successfully done for several AE features. The study aims to showcase an experimental system that detects the behaviour of capsules in the material. On that approach, precisely settled AE appliance carries the leading role.

The efficiently fixed experimental setup of two DIC cameras, eight AE sensors, and a CMOD device may be applied to hereby confirm healing recovery when after curing crack reopens.

## Figures and Tables

**Table 1:** DIC test set-up features.

Hardware setup	
2 CCD-cameras	AVT Stingray
Lenses	23 mm
Aperture	3.4
Software setup	
Resolution	2456 × 2058
Area of interest	100 × 100 mm—20 px/mm
Subset	27 × 27
Subset spacing	5
Strain field size	15
Testing setup	
Time capture	Every 3 seconds or Δload = 0.1 kN
Average speckle diameter	2.00 mm

**Table 2:** AE testing set-up features.

Analog filter	
Lower	100 kHz
Upper	2 MHz
Waveform setup	
Sample rate	1 MSPS
Pretrigger	256
Length	1 k
Timing setup	
PDT	100 *μ*s
HDT	200 *μ*s
HLT	500 *μ*s
Max duration	1000 *μ*s

**Table 3:** AE localization setup.

Wave velocity	4.000 m/sec
Event definition time	150 mm
Event lockout time	180 mm
Overcal time	20 mm
Hits/event	min: 4, max: 8
Max iterations	256
